# Dental Caries and Oral Health in Children—Special Issue

**DOI:** 10.3390/children8080674

**Published:** 2021-08-03

**Authors:** Santosh Kumar Tadakamadla, Gianluca Martino Tartaglia

**Affiliations:** 1 School of Medicine and Dentistry & Menzies Health Institute Queensland, Griffith University, Gold Coast 4214, Australia; 2 UOC Maxillo-Facial Surgery and Dentistry, Fondazione IRCCS Ca’ Granda, Ospedale Maggiore, Policlinico, 20122 Milan, Italy; gianluca.tartaglia@unimi.it; 3 Department of Medicine, Surgery and Dentistry, Fondazione IRCCS Ca’ Granda, Ospedale Maggiore, Policlinico, University of Milan, 20122 Milan, Italy

Oral diseases still pose a significant health burden affecting over 3.5 billion people worldwide. Dental caries is a major contributor to the global burden of oral diseases. Particularly, over half a billion children worldwide experience untreated caries in deciduous teeth [1] which could significantly impact their quality of life [2]. Dental caries is the most prevalent preventable disease that is multi-factorial, with diet and oral hygiene playing an important role in its prevention [3]. In children, oral conditions and behaviors are determined by multi-level factors that operate at the child-, family-, and community-level [4].

This special issue offers a selection of original articles and systematic reviews about the burden of oral diseases and behaviors, their determinants, the impact of these conditions on quality of life, and preventive and therapeutic approaches for their prevention and treatment in children. We have a variety of publications: two systematic reviews, one case report, and seven original research manuscripts.

Three articles were related to dental caries and its determinants. Even though the number of people with untreated oral conditions is decreasing in some countries, dental caries is still highly prevalent in other countries. For example, a study from Mexico demonstrated that over half of 6–12-year-old school children from agricultural manual worker households had caries in either or both dentitions, and a considerable proportion were untreated lesions. However, the authors noted that the prevalence levels in their study were lower than other reports from Mexico in similar age groups [5]. Another paper from Mexico quantified the association between self-reported dental caries and socioeconomic indicators in a national sample of Mexican children. Their findings suggested socioeconomic inequalities in self-reported dental caries in Mexican schoolchildren. Nevertheless, not all socioeconomic indicators correlated significantly with the outcome [6]. A systematic review and meta-analysis focused on the association between the most common vitamin D receptor polymorphisms (ApaI, FokI, TaqI, BsmI, and BglI) and the risk of dental caries in children. The review found that none of the polymorphisms was associated with the risk of dental caries, except for the FokI (rs10735810) polymorphism, with the f allele and ff genotype of this polymorphism exhibiting a protective role in the occurrence of dental caries [7].

Two studies were related to dental service utilization. Representative data from a large child population were used by Tiwari et al. (2021) to explore the association between the medical well-child visit (WCV) with preventive dental visit (PDV) among adolescents from 13 states in the US. More PDVs were made by children who participated in WCVs. Girls, Hispanics, and those aged 5–9 years had higher PDVs after a WCV [8]. Another article explored the predictors of unmet dental treatment needs and patterns of dental service utilization in adolescents in the Kingdom of Lesotho, Southern Africa. This study showed that only 14% of adolescents had seen a dental professional within the previous two years. They found that not having dental education and access to a regular dentist were the strongest predictors of not visiting a dentist last year [9].

A study from Australia evaluated an outreach mobile dental service targeted at Australian children with low household income. The authors found that the program reduced mean costs, restorations, and extractions in the subsequent year [10]. Oral diseases could significantly impact the quality of life in children; research on oral health-related quality of life (OHRQoL) is limited from the developing world. Tadakamadla et al. introduced a cross-cultural adaptation of the Hindi translation of the Child Perception Questionnaire in India. The authors found their translated OHRQoL measure to be reliable and valid [11].

In this issue, we also had a good collection of articles related to malocclusion and orthodontic treatment procedures. Abate et al reported findings using retrospective data on Rapid Maxillary Expansion (RME) safety on temporomandibular joints of growing patients with Juvenile Idiopathic Arthritis (JIA). They found that usage of RME in these special needs children with maxillary constriction is safe when JIA is in the quiescent phase, similar to healthy children [12]. Children with special needs have a high burden of oral diseases than normal children. For instance, retrospective data from the same database (UOC Unità operativa Complessa Chirurgia Maxillo-Facciale e Odontostomatologia, Fondazione IRCCS Ca Granda, Ospedale Maggiore Policlinico, Department of Medicine, Surgery and Dentistry University of Milan) used by Abate et al. demonstrated that the caries prevalence in children with special needs was twice to that of general population. This was based on the data of a sample of 115 children attending the hospital over a three months period. Farronato et al. conducted a systematic review to evaluate the association between malocclusion and allergic rhinitis. They reported that no conclusive evidence could be drawn, though most of the selected studies found an increased prevalence of malocclusion in those with allergic rhinitis [13]. Supporting the wide focus of this special issue, a case report presented a comprehensive orthopaedic and orthodontic treatment in an 8-year-old child with Becker’s nevus syndrome, which led to Class III malocclusion. Treatment resulted in ideal alignment and occlusion and proper overjet and overbite [14].

We hope that the readers will find the diversity of the content published in this special issue interesting and valuable. We had systematic reviews and original research reports, ranging from population-based studies to patient-centered clinical studies evaluating the effectiveness of treatment and preventive regimens.
